# A Bioinformatic Profile of Gene Expression of Colorectal Carcinoma Derived Organoids

**DOI:** 10.1155/2018/2594076

**Published:** 2018-09-27

**Authors:** Peng A, Xinyi Xu, Chenglin Wang, Ling Ye, Jing Yang

**Affiliations:** ^1^State Key Laboratory of Oral Diseases, West China School of Stomatology, Sichuan University, Chengdu 610041, Sichuan, China; ^2^National Clinical Research Center for Oral Diseases & Department of Cariology and Endodontics, West China Hospital of Stomatology, Sichuan University, Chengdu 610041, Sichuan, China

## Abstract

Colorectal carcinoma is one of the common cancers in human. It has been intensely debated whether the* in vitro* cancer cell lines are closely enough for recapitulating the original tumor in understanding the molecular characteristic of CRC. Organoid as a new* in vitro* 3D culture system has sprang out in CRC study for the capability in reviving the original tissue. The aim of this study is to profile the gene expression of CRC organoid. The gene expression GSE64392 was from GEO database contained 20-patients-derived 37 organoid samples, including 22 colorectal tumor organoid samples and 15 paired healthy samples. Gene ontology (GO) and Kyoto Encyclopedia of Genes and Genomes (KEGG) were applied for classifying differentially expressed genes (DEGs). Protein interaction among DEGs was analyzed by Search Tool for the Retrieval of Interacting Genes (STRING) and Cytoscape software. In total, 853 gene sequences were identified. GO analysis revealed that DEGs were extensively involved in various biological process (BP), like proliferation, cell cycle, and biosynthesis. KEEG pathway analysis showed that WNT, MAPK, TGF-*β*, SHH, ECM-receptor interaction, and FGF pathways were altered. DEGs which were identified with protein interactions were major response for extracellular matrix organization and the GPCR pathway. In conclusion, our study profiled the DEGs in CRC organoids and promotes our understanding of the CRC organoids as a new model for colorectal cancer research.

## 1. Introduction

Colorectal carcinoma (CRC) is one of the major cancers and is a contributor to cancer mortality and mobility in human. A variety of studies have revealed critical mutations of genes and the dysregulation of signaling pathways is important for the development of CRC [1]. Nevertheless, like other cancers, CRC presents the instability in genome, which usually leads to the diversity of cancer cell phenotype [1]. The genomic instability consisting of gene mutation and chromosomal hyperchange has been investigated as an important contributor of CRCs [1, 2]. To date, cancer cell lines are still mainly used in tumor research, as the accessibility and ease in manipulation [3], whereas cancer cell lines can be representative of tumors as an* in vitro* system is controversial [4, 5]. Recently, the Cancer Cell Line Encyclopedia (CCLE) characterized nearly 1,000 cancer cell lines via larger-scale genomic application [6]. Most cancer cell lines exhibited a relatively positive correlation in representing the original tumors from which cancer cell lines were derived. However, most cancer cell lines were derived from highly aggressive and fast growing tumor [3]; they tend to possess more genomic alterations than primary tumor, leading to be partial in representing the initiation or development of tumor [3]. Cancer cell line apparently limits in representing clinical attributes, like diagnosis, drug response, and treatment. A recent developed 3D culture system, organoid technology, demonstrated the maintenance of primary crypt physiology [7]. Then a long-term culture system was established for human intestinal and colonic epithelium organoid, indicating the application in development, pharmacology, and tumorigenesis of colon [8]. In addition, a large scale sequencing has been performed to characterize the developmental lineage tree on the organoid platform, reviving several features of normal mouse development [9]. Most recently, an established organoid bank of CRC patients resembled the primary tumor tissue physiologically [10]. Moreover, the genomic feature of tumor organoid mimics the primary tissue extensively [10]. Thus, analyzing the gene expression profile and the interaction of differentially expressed genes (DEGs) network of CRC organoid is crucial for understanding the biomedical application of organoid technology in CRCs and sustains that organoid is promising in personalized CRC therapy.

In this study, we analyzed gene expression profiles of healthy and tumor organoids of CRC patients with the GEO2R supported by Gene Expression Omnibus (GEO, http://www.ncbi.nlm.nih.gov/geo/). Subsequently, the DEGs were subjected to DAVID, to perform the gene ontology (GO) and pathway enrichment analysis. Then, we investigated the protein interaction among the DEGs. Our study may provide insight of organoids derived from patients as a potential 3D system for investigating the development of CRCs.

## 2. Materials and Methods

### 2.1. Microarray Data

The gene expression array of patients-derived healthy and tumor organoids GSE64392 was from GEO database. GSE64392, which was based on Affymetrix Human Gene 2.0 ST Arrays, was submitted by Marc van de Wetering et al. The GSE64392 dataset contained 37 organoid samples derived from 20 patients, including 22 colorectal tumor organoid samples and 15 paired healthy samples.

### 2.2. Gene Expression Profile Analysis

Differentially expressed genes were analyzed with the GEO2R, which accompanies with the GEO dataset and is supported by GEO database and available at https://www.ncbi.nlm.nih.gov/geo. Data were analyzed with default parameters. Genes with Log2-fold change between tumor and healthy samples ≥1 or ≤-1 were classified as differentially expressed genes (DEGs) and the adjusted P-value (adj.P.Val) < 0.05 was considered as statistical significance.

### 2.3. Gene Ontology and Pathway Analysis

Database for Annotation, Visualization, and Integrated Discovery (DAVID: https://david.ncifcrf.gov/) is a web application integrated various annotation sources including Gene ontology (GO) and Kyoto Encyclopedia of Genes and Genomes (KEGG) and is essential for the interpretation of high-throughput datasets. DAVID was applied for analyzing the enrichment of GO and KEGG pathway of DEGs. P<0.05 was considered as statistical significance.

### 2.4. Protein-Protein Interactions (PPIs) Analysis

Search Tool for the Retrieval of Interacting Genes (STRING) is a web-accessible database of protein-protein interactions (PPIs). STRING (version 10.5) currently covers 9′643′763 proteins from 2′031 organisms, including Homo sapiens. To evaluate the protein associations among DEGs, we mapped the DEGs to STRING; interactions with combined score ≥ 0.4 (medium confidence) were considered significant. Cytoscape (3.5.1) was used to visualize the interaction network. The plugin Molecular Complex Detection (MCODE) was performed to screen the network, with the MCODE score >3. Pathway enrichment was analyzed for clusters; P<0.05 was considered as significant difference.

## 3. Results

### 3.1. Differentially Expressed Genes

**Figure 1:**
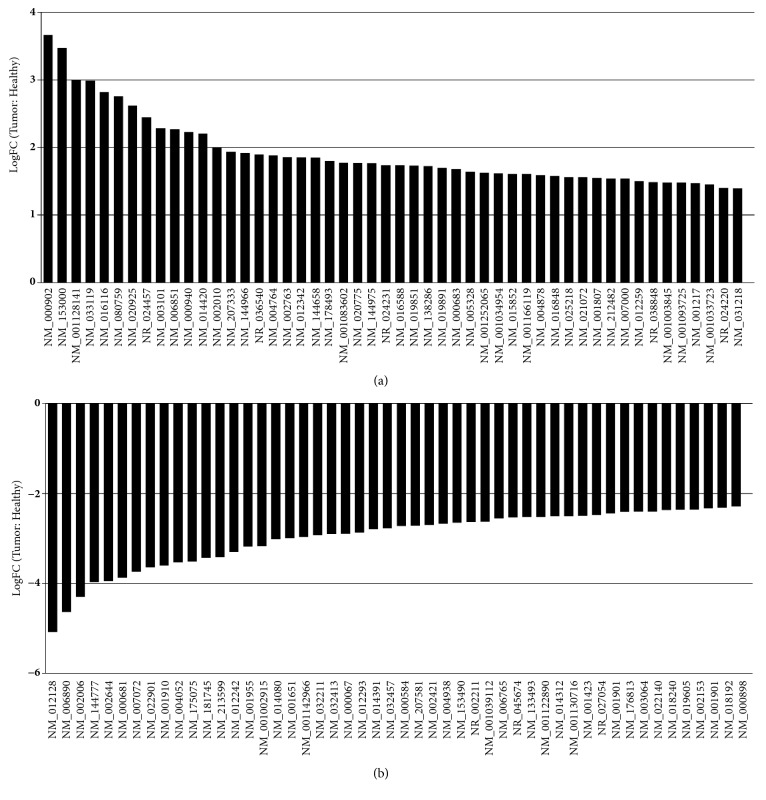
The top 100 differentially expressed genes (DEGs), 50 upregulated genes (a) and 50 downregulated genes (b).

Tissue derived organoids profoundly preserved the basic morphology and organization of primary tissues [7, 8, 10]. Moreover, tumor derived organoids profoundly revealed the genomic features of primary tumors [10]. Marc van de Wetering et al. compared the transcriptome profile of organoids with paired tumor tissues from nine patients. The gene expression profile of organoids displayed a high correlation (Pearson correlation 0.918 ± 0.040) with original paired biopsies, suggesting that organoids successfully recaptured the primary tumors on the scale of gene expressions [10]. Thus, it is grounded to analyze the organoids-based transcriptome profile. In this study, the total 37 organoid samples consisting with 22 tumors and 15 healthy samples were analyzed. Based on the GEO2R analysis, 853 gene sequences were identified. Only genes with GeneBank accession number according to National Center for Biotechnology Information (NCBI) database were listed as differentially expressed genes. Thus, a total of 405 genes were classified, 100 genes were upregulated in tumor organoid samples, and 305 genes were downregulated (Data not shown). The expression of top 50 upregulated and top 50 downregulated genes is listed (Figure 1).

### 3.2. Gene Ontology (GO) Enrichment Analysis

GO analysis was performed with the DAVID web application. Biological processes (BP) essential for the tumorigenesis exhibited the extensive mRNA expression change in tumor organoid samples. We grouped cell cycle, cell proliferation, and growth to the capability of tumor for doubling its population; both upregulated and downregulated genes enriched in tumor population process (Table 1). In addition, in tumor samples gene expression alteration was found enriched in metastasis and angiogenesis which are characters of cancer [11] (Table 1). Upregulated genes also enriched in the process of biosynthesis. Processes which are important for the survival of cancer cells including cell death evasion, inflammation process, and immune system all exhibited the genes downregulation (Table 1). In addition, downregulated genes showed enrichment in extracellular matrix organization, homeostasis, and secretion process. For cell component (CC), hyperexpressed genes only enriched in the plasma membrane. However, genes those downregulated in tumor samples were found in various aspects of cells, which could be mainly grouped to extracellular part, cell-cell communication (cell junction), plasma membrane, and cytoplasm organelles (Table 2). For molecular function (MF), overexpressed genes exhibited significant enrichments in nucleotide binding, including DNA binding and RNA polymerase activity (Table 3). Genes exhibiting the decrease in mRNA expression dramatically enriched in signaling molecular binding, including ion binding and ligand-receptor binding (Table 3).

### 3.3. KEGG Pathway Analysis

In the work by Marc van de Wetering et al., it is presented that the mutation rates per Mb of tumor organoids exhibited the similarity of paired biopsies. Mutations in organoids were predominantly CpG to T transitions, consistent with original tumor tissues. Moreover, somatic variants within the coding regions in organoids were highly concordant with the corresponding biopsies for both hypermutated and nonhypermutated patients (median = 0.88 frequency of concordance, range 0.62–1.00). Furthermore, combine the analysis of somatic copy number alterations (SCNAs) and single nucleotide variants (SNVs) to infer Cancer Fractions (CCF) between tumor organoids and biopsies, revealing that CRC driver mutations were maintained in organoids and most commonly altered genes and were all represented in organoids, including* APC, TP53, KRAS, PIK3CA, FBXW7*, and* SMAD4*. These results suggested that there were no distinct CRC driver mutations in organoids which may lead to the variation in pathways. Next, in order to identify the pathway enrichment of DEGs in tumor organoid samples, we investigated the KEGG pathway enrichment of DEGs using DAVID (Table 4). Thus, alteration of gene expression can be found in well-defined CRC related pathway including WNT, MAPK, and TGF-*β* pathways [1, 2]. We also grouped SHH, ECM-receptor interaction, and FGF signaling pathways. Hyperexpressed genes were also found to regulate cell adhesion. Interestingly, three upregulated genes in colorectal cancer organoid samples were also associated with basal cell carcinoma. Repressed genes were found to be tightly associated with Rap1 signaling and regulating Pantothenate and CoA biosynthesis, cytoskeleton, and renin-angiotensin system. In addition, downregulated genes were indicated to contribute the formation of bladder cancer in human.

### 3.4. Protein Interaction Network Analysis

**Figure 2:**
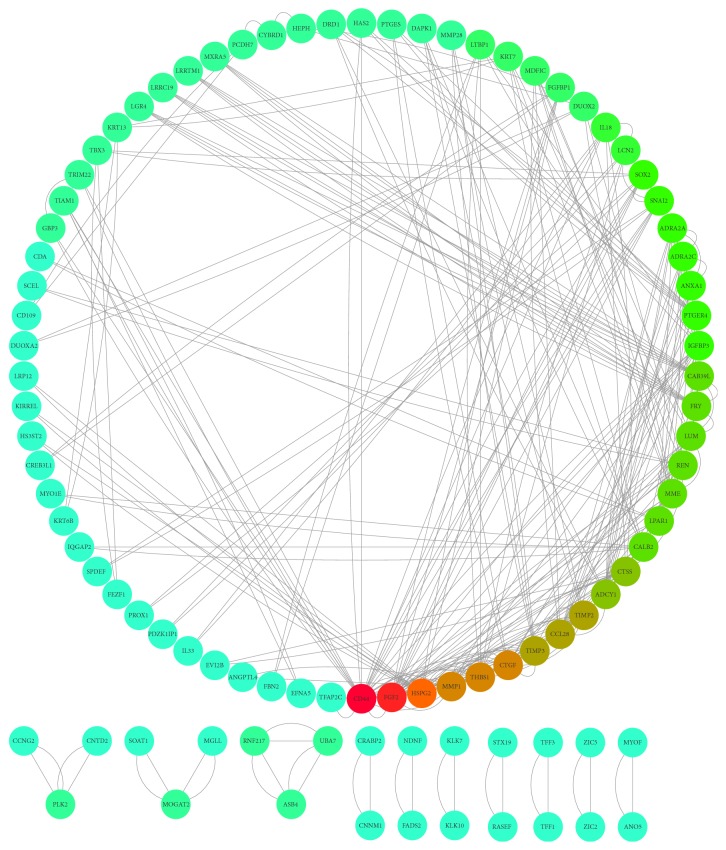
The protein-protein interaction (PPI) network of differentially expressed genes (DEGs).

**Figure 3:**
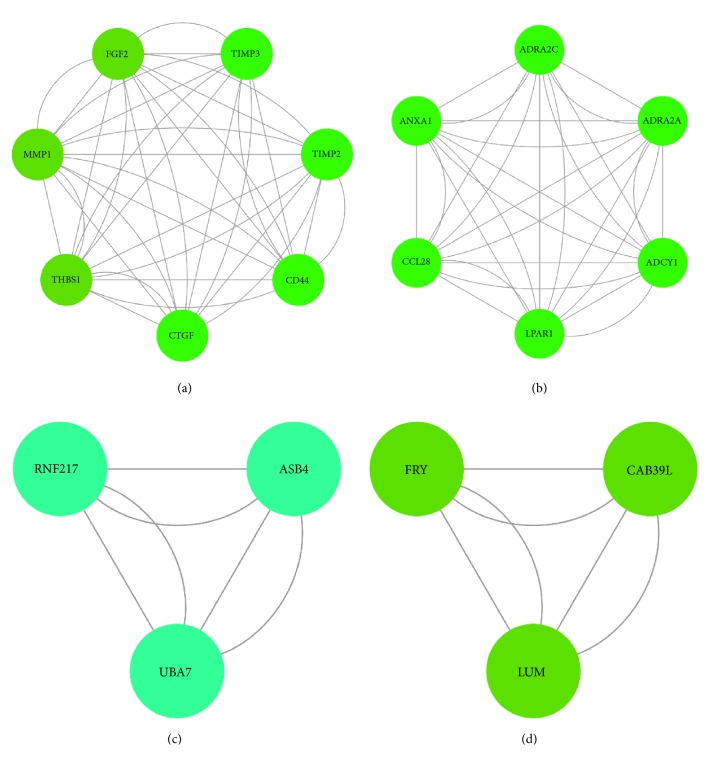
Top 4 modules of the protein-protein interaction (PPI) network were listed.

Protein-protein interactions (PPIs) are critical for the signaling transduction and the biological process. Thus, to investigate the protein interactions among the DEGs, we screened the protein interaction within the STRING database. Nodes with combined score ≥ 0.4 (medium confidence) were subjected to Cytoscape to visualize the protein interaction (Figure 2). Total nodes were subjected to analyze the interaction modules by using MCODE. Four modules were extracted from the network (Figures 3(a)~3(d)). Pathway enrichment analysis of modules showed that proteins were mainly involved in extracellular matrix organization and the GPCR signaling pathway.

## 4. Discussion

Colorectal cancer development is a complex process of accumulation of genetic mutations, epigenetic alteration, and dysregulation of signaling pathways [1]. Despite numerous progress has been made in elucidating the molecular mechanism of the development of CRC, the underlying mechanism remains vagueness. Cancer cell lines have been applied as the main workforce for cancer research [3, 6]. Nevertheless, like other tumors, CRC exhibits a high genomic instability which contributes to the various phenotypes and pathologies of CRCs [2]. Cancer cell lines limit in the capability of resembling the characteristics of the primary tumor tissue. Studies have reported the difference in gene expression and genomic alteration between cell line and tumors [4, 5, 12, 13]. Resulting from the identification of Lgr5+ stem cell in intestine and colon [14], a new 3D in vitro system, organoid [7], has made great impact on the colorectal research [10, 15, 16]. As organoids resemble the characteristics of primary tissue, organoids have been widely applied for studying organ development, tissue homeostasis, tumorigenesis, and disease [17–19]. As a promising* ex vivo *3D culture system for studying CRC, profiling genes expression level of CRC organoids is important for understanding the development of CRC. In the present study, we analyzed the data from GSE64392 and identified 100 upregulated genes and 305 downregulated genes in tumor organoids. DEGs were identified to involve in main capabilities of cancer, including cell renew, metastasis, angiogenesis, and cell death escaping. By analyzing the protein interaction of DEGs, we classified genes that may provide new insight for understanding the development of CRC.

In order to have a better view about the function of DEGs, GO analysis and KEEG pathway were performed. Upregulated genes were mainly involved in biological processes consisting of cell cycle, the cell proliferation controlling, tumor metastasis, and angiogenesis, which are essential for the sustentation of tumor [11]. For downregulated genes, in addition to involving the retaining of tumor, genes were also accumulated in biological processes which serves as barriers to cancer [11]. For example, programmed cell death, apoptosis, which is the most important physiologic processes in body to eliminate deteriorated cells [11], was another downregulated gene-enriched biological process. Moreover, downregulated genes were also found to participate in the following process, like extracellular matrix (ECM) organization, secretion process, immune-response, and homeostasis, alteration of which is acquired by cancer to escape the tethering of surrounding tissue and the chasing of immune system [11]. Organoid culture system has been proved to recapitulate the* in vivo* counterpart [20]; specifically, established CRC organoid cultures displayed a highly agreement with the primary tumor tissue, in genomic mutation, chromosomal alterations, and epigenetic modifications [1, 2, 10]. The KEGG pathway enrichment analysis revealed that genes in WNT pathway are upregulated, including* AXIN2, DKK4,* and* NKD1*, which are target of WNT/*β*-catenin signaling pathway and also antagonize WNT pathway. In addition, LEF1, which activates gene transcription in the axis of WNT signaling, was upregulated as well. The hyperexpression of WNT target genes corresponds with the hyperactivated WNT pathway in primary CRC and CRC cancer cell lines, most likely resulting from the biallelic inactivation mutation of* APC, FBXW7, AXIN2,* and* FAM123B *or the activating mutations in* CTNNB1* [2, 6]. Besides, overexpressed genes were identified to involve in other pathways, including FGF signaling, SHH signaling, and that are commonly found in various types of cancers [6, 21–23]. What is more, upregulated genes were related to cell-cell adhesion, alteration of which is widely reported in cancer [11, 24]. For downregulated genes, besides carcinoma common pathways, altered genes were identified in Rap1 pathway, which regulates various cancer tightly related biological processes, including angiogenesis control, cell movements, intercellular interaction, and cell expansion [6, 25]. Genes associated with CoA synthesis were also identified hypoexpressed, indicating the alteration of metabolism in CRCs [26, 27]. Also, downregulated genes were classed in regulating cytoskeleton, this is correlated with the dysregulation of signaling transduction intracellularly. Additionally, downregulated genes were identified in renin-angiotensin system which is an important pathway in regulating plasma sodium concentration and blood pressure, indicating that the development of CRCs may affect the homeostasis via the hormone system. Surveying alterations of these pathways may help us to understand the development of CRCs.

Protein interactions are essential for the signaling transduction within cells and communicating intercellularly and environmentally. The protein interaction module analysis of DEGs revealed that, in tumor organoid samples, PPIs were identified and enriched in extracellular matrix organization, GPCR signaling pathway. GPCRs are the largest receptor in eukaryotes; they are coupled with G proteins triggering signaling cascades to regulate various physiological processes [28]. The alteration of GPCRs signaling pathway, in cancer cells, promotes the proliferation, angiogenesis, and metastasis, aids to escape from apoptosis, and sustains the survive. Moreover, PPIs were also identified to involve in other signaling pathways, including AMPK signaling, mTOR signaling, and DNA damage, which are frequently altered in CRCs [2].

In conclusion, our study shows a bioinformatic analysis of differentially expressed genes in CRCs organoids. The study supports the fact that organoid technology as a promising in vitro 3D system recapitulates the property of tumors. Our study will encourage the exploration of the application of organoid as a more convenient resource for CRC therapy studies.

## Figures and Tables

**Table 1:** Gene ontology (GO) enrichment analysis of differentially expressed genes (DEG) in tumor organoids. Genes' enrichment of biological processes (BP). EMT: Epithelial-mesenchymal transition; ECM: Extracellular matrix.

**Category**	**Group**	**Term**		**GenesCount**	**GenesCount ratio of Total genes**	**P-Value**	**Benjamini**
GOTERM_BP_ALL	Upregulated	**Population**	Cell cycle (GO:0051726)	10	10	4.80E-02	3.20E-01
			Cell proliferation (GO:0042127)	20	20	1.40E-04	4.80E-03
			Growth (GO:0040007)	16	16	3.90E-05	2.10E-03
		**Metastasis**	Cell migration (GO:0016477)	15	15	1.70E-03	2.90E-02
			Cell motility (GO:0048870)	16	16	1.80E-03	3.00E-02
			Locomation (GO:0040011)	17	17	2.70E-03	4.00E-02
			EMT (GO:0001837)	8	8	7.40E-07	2.00E-04
			Cell adhesion (GO:0007155)	15	15	3.50E-02	2.70E-01
		**Agiogenesis**	Agiogenesis (GO:0001525)	8	8	4.00E-03	5.50E-02
			Vasculogenesis (GO:0001570)	4	4	5.10E-03	6.40E-02
		**Others**	Biosynthetic process (GO:0009889)	37	37	2.90E-04	7.90E-03
	Downregulated	**Metastasis**	Cell migration (GO:0016477)	34	11.5	2.70E-04	1.70E-02
			Cell motility (GO:0048870)	36	12.2	5.10E-04	2.50E-02
			Locomotion (GO:0040011)	39	13.2	8.70E-04	3.50E-02
			Localization of cell (GO:0051674)	36	12.2	5.10E-04	2.50E-02
			Cell adhesion; GO:0007155	37	12.5	1.50E-02	2.10E-01
		**Evading Cell death**	Cell death (GO:0008219)	49	16.6	2.20E-04	1.50E-02
			Apoptosis (GO:0006915)	44	14.9	4.60E-04	2.30E-02
		**Population**	Growth (GO:0040007)	27	9.1	1.30E-03	4.30E-02
			Cell proliferation (GO:0008283)	42	14.2	5.00E-03	1.00E-01
		**Inflammatory response**	Chronic inflammatory response (GO:0002544)	3	1	4.10E-02	3.60E-01
			Inflammatory response (GO:0006954)	19	6.4	6.10E-03	1.20E-01
		**Agiogenesis**	Agiogenesis (GO:0001525)	12	4.1	4.10E-02	3.60E-01
		**Others**	ECM orgnazation (GO:0030198)	20	6.8	5.10E-07	2.20E-03
			Homeostatic process (GO:0042592)	35	11.8	2.50E-02	2.80E-01
			Secretion (GO:0046903)	33	11.1	1.50E-04	1.20E-02
			Immune system process (GO:0002376)	50	16.9	1.50E-02	2.10E-01

**Table 2:** Gene ontology (GO) enrichment analysis of differentially expressed genes (DEGs) in tumor organoids. Genes' enrichment of cell component (CC).

**Category**	**Group**	**Term (GO)**	**GenesCount**	%	**P-Value**
GOTERM_CC_ALL	Upregulated	Plasma membrane part (GO:0044459)	21	21	2.20E-02
	Downregulated	Extracellular region part (GO:0044421)	111	37.5	1.90E-13
		Extracellular region (GO:0005576)	120	40.5	1.00E-11
		Extracellular space (GO:0005615)	52	17.6	4.00E-09
		Extracellular exosome (GO:0070062)	76	25.7	1.10E-07
		Vesicle (GO:0031982)	93	31.4	1.20E-07
		Extracellular vesicle (GO:1903561)	76	25.7	1.30E-07
		Extracellular organelle (GO:0043230)	76	25.7	1.40E-07
		Proteinaceous extracellular matrix (GO:0005578)	21	7.1	4.50E-07
		Extracellular matrix component (GO:0044420)	12	4.1	3.10E-06
		Extracellular matrix (GO:0031012)	24	8.1	4.20E-06
		Cell periphery (GO:0071944)	111	37.5	1.40E-05
		Plasma membrane (GO:0005886)	108	36.5	2.50E-05
		Plasma membrane part (GO:0044459)	65	22	2.90E-05
		Plasma membrane region (GO:0098590)	31	10.5	5.60E-05
		Basement membrane (GO:0005604)	9	3	8.10E-05
		Microvillus (GO:0005902)	8	2.7	1.10E-04
		Cell surface (GO:0009986)	26	8.8	1.80E-04
		Intrinsic component of plasma membrane (GO:0031226)	43	14.5	6.30E-04
		Apical part of cell (GO:0045177)	15	5.1	1.00E-03
		Integral component of plasma membrane (GO:0005887)	41	13.9	1.00E-03
		Membrane part (GO:0044425)	130	43.9	1.20E-03
		Actin-based cell projection (GO:0098858)	10	3.4	1.40E-03
		Intrinsic component of membrane (GO:0031224)	113	38.2	1.40E-03
		Apical plasma membrane (GO:0016324)	13	4.4	1.40E-03
		Anchored component of membrane (GO:0031225)	9	3	2.30E-03
		Integral component of membrane (GO:0016021)	110	37.2	2.40E-03
		Membrane raft (GO:0045121)	12	4.1	2.80E-03
		Membrane microdomain (GO:0098857)	12	4.1	2.90E-03
		Brush border (GO:0005903)	7	2.4	4.00E-03
		Cluster of actin-based cell projections (GO:0098862)	8	2.7	5.40E-03
		Membrane region (GO:0098589)	13	4.4	6.60E-03
		Endomembrane system (GO:0012505)	75	25.3	8.70E-03
		Basolateral plasma membrane (GO:0016323)	9	3	1.10E-02
		Membrane (GO:0016020)	158	53.4	1.90E-02
		Caveola (GO:0005901)	5	1.7	2.30E-02
		Microvillus membrane (GO:0031528)	3	1	3.10E-02
		Plasma membrane raft (GO:0044853)	5	1.7	3.30E-02
		Cytoplasmic vesicle (GO:0031410)	28	9.5	4.00E-02
		Intracellular vesicle (GO:0097708)	28	9.5	4.10E-02
		Spanning component of membrane (GO:0089717)	2	0.7	4.40E-02
		Spanning component of plasma membrane (GO:0044214)	2	0.7	4.40E-02
		Actin cytoskeleton (GO:0015629)	13	4.4	4.50E-02
		Cytoplasmic, membrane-bounded vesicle (GO:0031410)	26	8.8	4.50E-02

**Table 3:** Gene ontology (GO) enrichment analysis of differentially expressed genes (DEGs) in tumor organoids. Genes' enrichment of molecular function (MF).

**Category**	**Group**	**Term**	**Count**	%	**P-Value**
GOTERM_MF_ALL	Upregulaged	sequence-specific double-stranded DNA binding (GO:1990837)	14	14	3.40E-05
		transcription regulatory region sequence-specific DNA binding (GO:0000976)	13	13	9.60E-05
		double-stranded DNA binding (GO:0003690)	14	14	9.80E-05
		transcription factor activity, sequence-specific DNA binding (GO:0003700)	16	16	5.90E-04
		nucleic acid binding transcription factor activity (GO:0001071)	16	16	6.00E-04
		transcription regulatory region DNA binding (GO:0044212)	13	13	6.50E-04
		regulatory region DNA binding (GO:0000975)	13	13	6.70E-04
		regulatory region nucleic acid binding (GO:0001067)	13	13	6.80E-04
		sulfur compound binding (GO:1901681)	7	7	8.30E-04
		RNA polymerase II transcription factor activity, sequence-specific DNA binding (GO:0000981)	11	11	1.10E-03
		transcription factor activity, RNA polymerase II core promoter proximal region sequence-specific binding (GO:0000982)	8	8	1.20E-03
		receptor binding (GO:0005102)	17	17	1.20E-03
		RNA polymerase II core promoter proximal region sequence-specific DNA binding (GO:0000978)	8	8	1.50E-03
		transcriptional repressor activity, RNA polymerase II transcription regulatory region sequence-specific binding (GO:0001227)	6	6	1.50E-03
		sequence-specific DNA binding (GO:0043565)	14	14	1.60E-03
		transcriptional repressor activity, RNA polymerase II core promoter proximal region sequence-specific binding (GO:0001078)	5	5	1.90E-03
		carbonate dehydratase activity (GO:0004089)	3	3	1.90E-03
		core promoter proximal region sequence-specific DNA binding (GO:0000987)	8	8	2.00E-03
		core promoter proximal region DNA binding (GO:0001159)	8	8	2.10E-03
		RNA polymerase II regulatory region sequence-specific DNA binding (GO:0000977)	10	10	2.10E-03
		RNA polymerase II regulatory region DNA binding (GO:0001012)	10	10	2.20E-03
		activating transcription factor binding (GO:0033613)	4	4	2.80E-03
		DNA binding (GO:0003677)	23	23	3.20E-03
		metal ion binding (GO:0046872)	32	32	3.70E-03
		ion binding (GO:0043167)	33	33	4.20E-03
		cation binding (GO:0043169)	32	32	4.40E-03
		RNA polymerase II activating transcription factor binding (GO:0001102)	3	3	1.40E-02
		kinase binding (GO:0019900)	8	8	2.30E-02
		hydro-lyase activity (GO:0016836)	3	3	2.50E-02
		carboxyl-O-methyltransferase activity (GO:0010340)	2	2	3.30E-02
		protein carboxyl O-methyltransferase activity (GO:0051998)	2	2	3.30E-02
		binding (GO:0005488)	75	75	3.80E-02
		protein kinase binding (GO:0019901)	7	7	4.00E-02
		heparin binding (GO:0008201)	4	4	4.00E-02
		enzyme binding (GO:0019899)	15	15	4.10E-02
		modified amino acid binding (GO:0072341)	3	3	4.40E-02
		carbon-oxygen lyase activity (GO:0016835)	3	3	4.70E-02
		alcohol binding (GO:0043178) )	3	3	4.80E-02
	Downregulated	calcium ion binding (GO:0005509	32	10.8	8.80E-08
		growth factor binding (GO:0019838)	11	3.7	2.10E-05
		laminin binding (GO:0043236)	5	1.7	9.50E-04
		NADH pyrophosphatase activity (GO:0035529)	3	1	2.10E-03
		phospholipid binding (GO:0005543)	14	4.7	2.50E-03
		collagen binding (GO:0005518)	6	2	2.60E-03
		cytokine activity (GO:0005125)	10	3.4	6.00E-03
		fibronectin binding (GO:0001968)	4	1.4	6.50E-03
		metal ion binding (GO:0046872)	80	27	7.60E-03
		extracellular matrix binding (GO:0050840)	5	1.7	7.80E-03
		sulfur compound binding (GO:1901681)	10	3.4	8.10E-03
		lipid binding (GO:0008289)	19	6.4	9.10E-03
		receptor antagonist activity (GO:0048019)	3	1	9.10E-03
		receptor binding (GO:0005102)	34	11.5	9.80E-03
		heparin binding (GO:0008201)	8	2.7	9.90E-03
		cation binding (GO:0043169)	80	27	1.00E-02
		calcium-dependent phospholipid binding (GO:0005544)	5	1.7	1.10E-02
		glycosaminoglycan binding (GO:0005539)	9	3	1.30E-02
		ion binding (GO:0043167)	82	27.7	1.30E-02
		enzyme inhibitor activity (GO:0004857)	13	4.4	1.40E-02
		receptor inhibitor activity (GO:0030547)	3	1	1.80E-02
		nucleotide diphosphatase activity (GO:0004551)	3	1	2.30E-02
		transforming growth factor beta binding (GO:0050431)	3	1	2.30E-02
		metalloendopeptidase inhibitor activity (GO:0008191)	3	1	2.30E-02
		protein homodimerization activity (GO:0042803)	19	6.4	2.40E-02
		binding (GO:0005488)	225	76	2.60E-02
		receptor regulator activity (GO:0030545)	4	1.4	3.20E-02
		actin binding (GO:0003779)	12	4.1	3.50E-02
		protein binding (GO:0005515)	175	59.1	3.60E-02
		interleukin-1 receptor antagonist activity (GO:0005152)	2	0.7	4.40E-02
		fibroblast growth factor binding (GO:0017134)	3	1	4.50E-02
		cytoskeletal protein binding (GO:0008092)	20	6.8	4.70E-02

**Table 4:** Kyoto Encyclopedia of Genes and Genomes (KEGG) pathway analysis of differentially expressed genes (DEGs) in tumor organoids.

Category	KEGG pathway	Genes Count	%	P-Value	Gene members
Upregulated	Wnt signaling pathway	5	5	3.20E-03	NM_012342, NM_004655, NM_014420, NM_001166119, NM_033119
	Basal cell carcinoma	3	3	2.50E-02	NM_004655,NM_001166119, NM_001083602
	Pathways in cancer	6	6	2.90E-02	NM_004655, NM_019851, NM_002010, NM_212482, NM_001166119, NM_001083602
	Adherens junction	3	3	4.00E-02	NM_001166119, NM_005985, NM_001034954

Downregulated	Rap1 signaling pathway	9	3	1.40E-02	NM_000899, NM_003253, NM_021116, NM_001992, NM_001962, NM_002006, NM_022970, NM_057159, NM_003246
	Bladder cancer	4	1.4	2.40E-02	NM_000584, NM_004938, NM_002421, NM_003246
	Pantothenate and CoA biosynthesis	3	1	3.00E-02	NM_006208, NM_005021, NM_004666
	Pathways in cancer	12	4.1	3.60E-02	NM_000584, NM_000899, NM_004991, NM_021116, NM_001200, NM_001992, NM_004938, NM_002006, NM_022970, NM_057159, NM_002421, NM_000958
	Regulation of actin cytoskeleton	8	2.7	4.10E-02	NM_006633, NM_003253, NM_001992, NM_002006, NM_022970, NM_001127663, NM_053025, NM_001112706
	Renin-angiotensin system	3	1	4.70E-02	NM_001150, NM_021804, NM_000537

## Data Availability

The data used to support the findings of this study are available from the corresponding author upon request.
